# MicroRNA-93 promotes proliferation and metastasis of gastric cancer via targeting TIMP2

**DOI:** 10.1371/journal.pone.0189490

**Published:** 2017-12-08

**Authors:** Hao Guan, Weiming Li, Yuanyuan Li, Jichang Wang, Yan Li, Yanan Tang, Shaoying Lu

**Affiliations:** 1 Department of Vascular Surgery, The First Affiliated Hospital of Xi’an Jiao Tong University, Xi’an, China; 2 Department of Translational Medicine Center, The Second Affiliated Hospital of Shaanxi University of Chinese Medicine, Xianyang, China; China Medical University, TAIWAN

## Abstract

MicroRNAs (miRNAs) are important regulators of pathobiological processes in various cancer. In the present study, we demonstrated that miR-93 expression was significantly up-regulated in gastric cancer tissues compared with that in matched normal mucosal tissues. High expression of miR-93 was significantly associated with lymph node metastasis and tumor-node-metastasis (TNM) stage. Functionally, ectopic expression of miR-93 promoted cell proliferation, migration, invasion, EMT phenotypes, and repressed apoptosis and G1 cell cycle arrest in vitro, and promoted tumor formation in vivo. We further identified that tissue inhibitor of metalloproteinase 2 (TIMP2) was a direct target of miR-93 by using luciferase reporter assay, qRT-PCR, and immunoblotting assay. Furthermore, knockdown of TIMP2 with specific siRNA showed similar oncogenic effects in gastric cancer cells with that transfected with miR-93 mimics. Our findings indicated that miR-93 serves as a tumor promoter in human gastric carcinogenesis by targeting TIMP2, suggesting that miR-93 might be a promising biomarker and therapeutic target for treatment of gastric cancer.

## Introduction

Gastric cancer is the fourth most common cancer and the second leading cause of cancer-related death in the world [1]. Although improvement in clinical outcomes and therapies has decreased the incidence and mortality trends of gastric cancer in most developed countries, the incidence and mortality rates are still high in low-resource areas, predominantly in China due to a lack of early diagnosis [2–3]. Patients with late stage of gastric cancer are incurable and have unfavorable 5-year overall survival rate of nearly 4% [4]. Therefore, investigation of molecular mechanisms underlying gastric cancer metastasis and identification of novel therapeutic targets are pivotal to improve prognosis and treatment of gastric cancer.

MicroRNAs (miRNAs) are a group of small non-protein-coding RNAs with 18–25 nucleotides sequences that regulate gene expression through binding to complementary sequences in the 3’-untranlated regions of the target mRNAs, influencing post-transcriptional repression and mRNA degradation [5]. miRNAs play important roles in cellular processes including cell proliferation, invasion and migration in variety of human cancers [6–7]. As such, miRNAs can directly influence tumorigenesis and progression of cancer.

By comparing the expression profiles of miRNAs in gastric cancer, we found that miR-93 was obviously upregulated in gastric cancer tissues compared with that in matched normal mucosal tissues. The aberrant expression of miR-93 have been also reported and which may act as oncogene or tumor suppressor in numerous types of cancer, including glioma, hepatocellular cancer, endometrial carcinoma and breast cancer. In glioma, miR-93 was found to facilitate cancer development by interacting with PI3K/AKT pathway [8]. The study in breast cancer showed that miR-93 was a new therapeutic and prognostic biomarker, which could restrain cell migratory capability and invasive potential of cancer cells [9]. In addition, miR-93 was found to act as an oncogene in hepatocellular carcinoma and was correlated with unfavorable clinicopathological characteristics of patients [10]. Moreover, miR-93 was a metastatic promoter and may accelerate the process of epithelial-mesenchymal transition (EMT) in endometrial carcinoma cells [11]. In the present study, we found that miR-93 was significantly up-regulated in gastric cancer tissues compared with that in matched normal mucosal tissues, and analyzed the correlations between miR-93 expression and the clinicopathologic features. Subsequently, we demonstrated that miR-93 played a role in promoting the cell proliferation, migration and invasion and induce EMT in gastric cancer cells. By using bioinformatics and luciferase reporter assay, it was revealed that TIMP2 was a direct target of miR-93 and could reverse oncogenic functions of miR-93 in gastric cancer. In conclusion, our findings highlighted the key roles of miR-93 in gastric carcinogenesis and progression, and provided a promising therapeutic target in gastric cancer.

## Materials and methods

### Cell lines and tissue samples

The human gastric cancer cells line, SGC-7901, MKN-28, BGC-823, MGC-803, MKN-45, human normal gastric mucosal epithelial cell line GES-1, and human embryo kidney epithelial cell 293T (HEK-293T) were purchased from the Cell bank Center, Shanghai Institute of Biochemistry and Cell Biology, Chinese Academy of Sciences (Shanghai, China). All cells were cultured in a humidified 5% CO_2_ atmosphere at 37°C, and incubated in PRMI1640 medium supplemented with 10% fetal bovine serum (GIBCO, Carlsbad, CA, USA),along with 100 U/mL penicillin and 100 μg/mL streptomycin. The cells were used for experiments when they were at exponential phase after checking the morphology. Gastric cancer tissues and adjacent non-tumor mucosae were derived from patients who underwent radical gastrectomy from January 2014 to December 2015 at the Department of Surgery, the First Affiliated Hospital of Xi’an Jiaotong University. None of patients received radiation or chemotherapy before surgery. All the tissues were collected in the same condition and were histopathologically verified carcinoma. Tissue fragments were immediately frozen in liquid nitrogen at the moment of surgery. The study was approved by the Ethics committee of Xi’an Jiaotong University, and all patients have signed a written informed consent for using the specimens.

### RNA isolation and quantitative RT-PCR

Total RNA were extracted from tissue specimens and cell lines using Trizol one-step method (Takara, Shiga, Japan). All the operations were performed under a clean condition according to the manufacturer’s instructions. OD260/OD280 ratio of isolated RNA samples ranging from 1.9–2.0 were considered good quality. Isolated RNA was used as a template to synthesize the cDNA. The miR-93 expression was measured by real-time PCR using TaqMan miRNAs Quantitation Kit (Applied Biosystems, Foster City, CA, USA). The date were analyzed using threshold cycle (Ct) value, and the mean Ct was evaluated from three independent PCRs. U6 snRNA was used as an internal control for miRNA quantitative PCR. GAPDH was used as an internal control for mRNA. All the primers sequences were listed in Table 1. Each experiment was performed at least three times.

**Table 1:** The sequence of primers used for qRT-PCR.

Gene	Name	Sequence(5’ to 3’)
Has-miR-93	miR-93-RT	GTCGTATCCAGTGCAGGGTCCGAGGTATTCGCACTGGATACGACCTACCT
	miR-93-F	AGGCCCAAAGTGCTGTTCGT
	miR-93-R	GTGCAGGGTCCGAGGT
U6	U6-RT	GTCGTATCCAGTGCAGGGTCCGAGGTATTCGCACTGGATACGACAAAAAT
	U6-F	CTCGCTTCGGCAGCACA
	U6-R	AACGCTTCACGAATTTGCGT
TIMP2	TIMP2-F	GAACATCAACGGGCACCAG
	TIMP2-R	TCCCTCCAGAACCCACAACC
GAPDH	GAPDH-F	CTCTGATTTGGTCGTATTGGG
	GAPDH-R	TGGAAGATGGTGATGGGATT
SiTIMP2	SiTIMP2-F	CTCTGATTTGGTCGTATTGGG
	SiTIMP2-R	TGGAAGATGGTGATGGGATT
E-cadherin	E-cadherin-F	TGCTGTTTCTGGTTTCTGTTGG
	E-cadherin-R	CCTTCTCCGTATTTCTCCTCCCT
N-cadherin	N-cadherin-F	TTTGGGGAGGGGTAAAAGTTC
	N-cadherin-R	AAGAAACAGGCCACCCCGTTT
Vimentin	Vimentin-F	CGGTTGAGACCAGAGATGGA
	Vimentin-R	TGCTGGTACTGCACTGTTGGT

### Transfection

MiR-93 mimics, inhibitor, and negative control were obtained from Ribobio (Guangzhou, China.) The TIMP2 siRNA and negative control were purchased from Sigma Aldrich (St. Louis, MO, USA). Cells were seeded in six-well plates for 24 h, and then transfected with miR-93 mimic or its inhibitor, TIMP2 siRNA, or their scrambled negative control by using Lipofectamine 2000 (Invitrogen, Carlsbad, CA, USA) following the manufacturer’s protocol.

### Cell viability assay

MTT assays was performed to detect cell proliferation capacity. Briefly, cells transfected with miR-93 mimics/inhibitor were seeded into 96-well plates at a cell density of 2×10^3^ cells per well. At 24, 48, 72 and 96 h after transfection, cells were stained with MTT (25 μL/well) and incubated for 4 h at 37°C. The absorbance was examined at 450 nm and the cell viability was calculated. Experiments for cell viability assay were repeated at least three times.

### Apoptosis assay

Cells were collected 48 h post transfection and then stained with 5 μL Annexin V-FITC and 5μL PI (BD Biosciences, USA) for 15 min. The stained cells were analyzed by flow cytometer according to the manufacturer’s instructions (FACSC alibur, BD, USA).

### Cell cycle analysis

48 h post-transfection of cells with miR-93 mimics, inhibitor or control (100nM), SGC-7901 and BGC-823 cells were collected and resuspended in phosphate buffered saline (PBS). For cell cycle assays, cells were fixed by 75% pre-cooled ethanol and stored at 4°C. Prior to detection, fixed cells were washed twice with PBS, disposed with RNase A (50 μg/ml), and stained with PI at 25°C for 30 min in the dark. The cell cycle was analyzed by flow cytomety (FACSC alibur, BD, USA). The cell percentage of the G0/G1, G2/M and S phases were calculated by using the Flowjo7.6.3 (Treestar).

### Transwell assay

For cell migration assays, 2×10^4^ transfected cells in serum-free medium were added into the upper separate compartment of transwell chamber (24-well insert, 8.0μm in pore size, Corning). Medium containing 10% FBS that placed into the bottom chamber was used as a chemoattractant. For cell invasion assays, transfected cells were seeded into the upper chamber of the transwell after adding diluted matrigel in 5% CO_2_ atmosphere at 37°C for half an hour. After 24h incubation for migration assays and 48h incubation for invasion assays at 37°C of 5% CO_2_ atmosphere, cells on the top surface of the filters that did not pass through the pores were removed from the upper chamber using a cotton swab, while cells on the bottom surface of the membrane that migrated or invaded through the pores were fixed with 4% paraformaldehyde for 30 min, and then stained with 0.1% crystal violet (Sigma). Images from 5 different fields were taken and counted under a microscope. Each experiment was performed in triplicate.

### Western blotting

Cells seeded in 6-well plates were harvested after intervention treatment and the cellular proteins were extracted by using the RIPA lysis buffer. Western blot analysis was performed according to manufacturer’s protocol. The protein concentration was quantified by using the BCA kit (Pierce, Rockford, IL, USA). 20–40μg proteins each sample were subjected to 10% SDS-PAGE and transferred to PVDF membrane. The membrane was blocked with Tris-based saline-Tween 20(TBST) containing 5% skim milk for 1 h at room temperature. The primary antibodies against TIMP2, MMP2, MMP9, E-cadherin, N-cadherin, Vimentin (1:2000, Santa Cruz Biotechnology, Santa Cruz, CA, USA) and GAPDH (1:2000, Cell Signaling Technology, Danvers, USA) were incubated with the membranes at 4°C for overnight. After three times washing by TBST, the secondary antibodies (1:2000, Abcam, Cambridge, UK) against related primary antibodies were incubated with membranes for 2 h at room temperature. The protein bands were visualized using electrochemiluminescence (ECL) and testing reagents (Amersham Biosciences Corp., Piscataway, NJ, USA).

### Immunohistochemical staining

20 days after injection of mice with gastric cancer cells, tumor tissues from nude mice were isolated and washed in PBS, and fixed with 4% paraformaldehyde and paraffin tissue slides (4 μm) were made. Sections were processed by microwave and wiped out their peroxidase activity. 0.5% Triton was used to break cell membranes. Before applying the primary antibodies, the step of antigen retrieval was carried out. The slides were incubated with rabbit anti-human TIMP2 antibody (1:200, Abcam, Cambridge, UK) for overnight at 4°C, and then incubated with secondary antibodies (1:500, Abcam, Cambridge, UK) for 2 h at 25°C. After slides were dyed by DBA (Sigma, USA) and counterstained by hematoxylin, slides were sealed for visualizing.

### Target prediction

The miRNA target genes and their fragments with action site were predicted using a variety of online analysis softwares, including TargetScan, miRanda, miRbase and PicTar algorithms. Kyoto Encyclopedia of Genes and Genomes (KEGG) was also used to detect the biological functions of the target gene.

### Luciferase reporter assay

To perform the dual-luciferase reporter gene assay, we designed a wild-type or mutant TIMP2 3’UTR luciferase reporter vector, the 3’UTR sequence of TIMP2 including the fragment with predicted binding site for miR-93 or mutant were cloned into the pGL3-promoter vector. The vector was co-transfected with miR-93 mimics/inhibitor or negative control using Lipofectamine 2000. PGL-TK vector expressing Renilla luciferase was served as the transfection control. After co-transfection for 48 h, Luciferase activities containing Renilla luciferase and firefly were evaluated using a Dual-Luciferase Reporter detection System (Promega, Madison, WI, USA) following the manufacturer’s instructions. For each assay, the luciferase activity was performed in triplicate.

### Tumor xenograft model

Female BALB/c nude mice (4 weeks-old) were purchased from the Medical Experimental Animal Center of Xi’an Jiao Tong University (Shannxi, China). All animal experiments were approved by the Animal Care Committee of First Affiliated Hospital, Xi’an Jiao Tong university School of medicine. For tumor growth research, 0.2 mL PBS containing 1×10^6^ cells of SGC-7901 transfected with miR-93 antagomir or scramble control (Ribobio, Guangzhou, China) were injected into right armpit of each nude mice after using ethanol and iodine to sterilize the insertion area. Tumor volume were measured by vernier caliper every three days. Mice were euthanized and tumors were separated, measured, photographed after observing three weeks.

### Statistical analysis

SPSS statistical software (SPSS, Chicago, IL, USA), version 18.0 was used to analyze data. Quantitative data were presented as mean ± standard deviation (X±SD). The comparison between data was calculated using Student’s t-test and one-way variance analysis. The correlation between miR-93 and TIMP2 expression levels was determined with Pearson’s correlation analysis. P<0.05 in all cases was set as statistically significant.

## Results

### MiR-93 is up-regulated in gastric cancer cells and clinical samples

**Fig 1:**
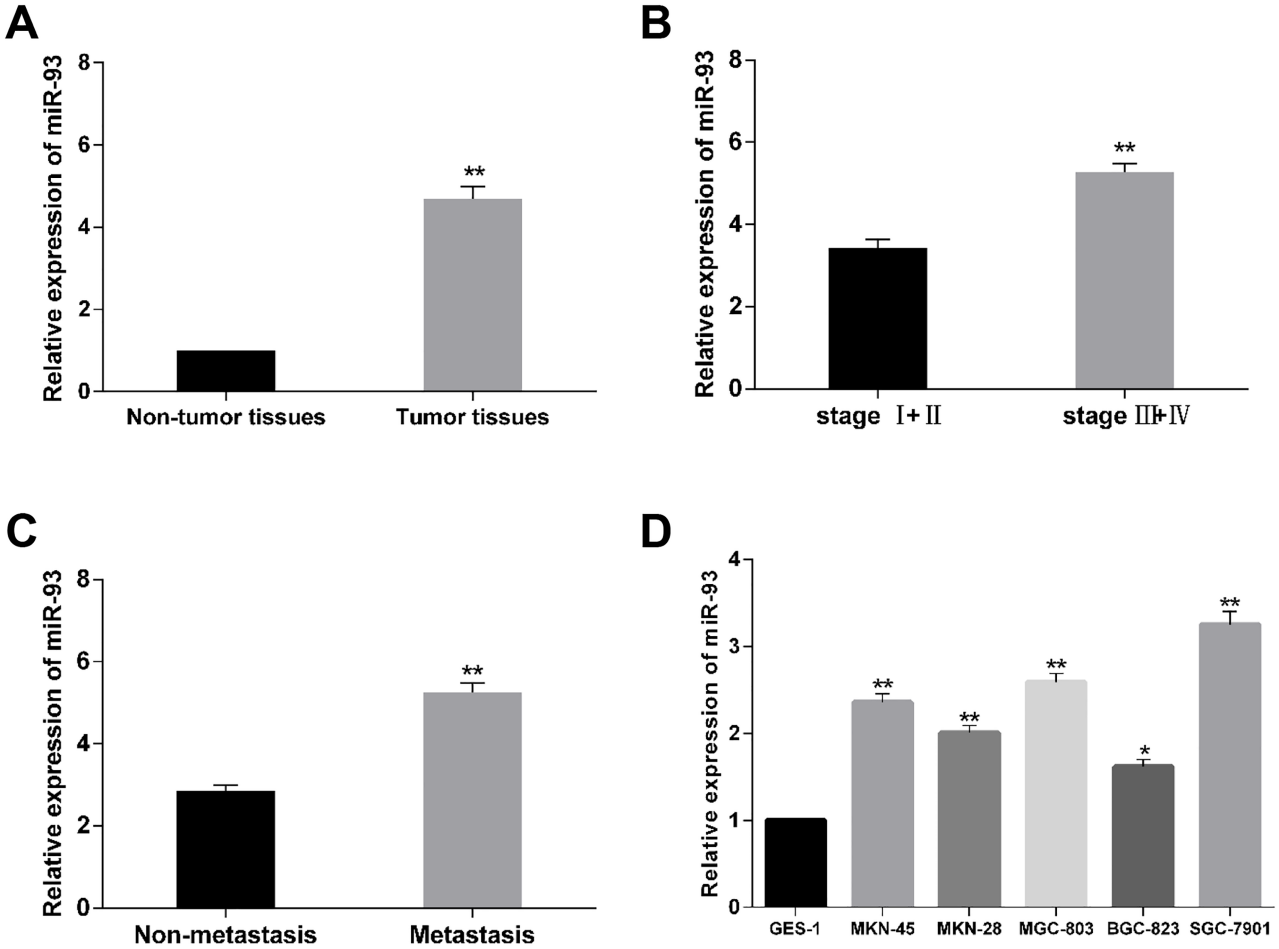
The expression level of miR-93 in gastric cancer clinical specimens and cell lines. (A) qRT-PCR result for the expression of miR-93 in gastric cancer tissues and matched adjacent normal tissues. (B) Relative expression level of miR-93 in patients at different clinical stages. (C) Relative expression level of miR-93 in patients with metastasis or without metastasis. (D) Relative expression level of miR-93 in gastric cancer cell lines relative to the normal human gastric epithelial cell line GES-1. *P<0.05, **P<0.01.

To validate the expression condition of miR-93 in human gastric cancer tissues, the miR-93 level in tumor tissues and paired adjacent normal tissues isolated from 70 patients was detected by using qRT-PCR. The results indicated that expression of miR-93 was significantly higher in gastric cancer tissues than in the adjacent normal tissues (Fig 1A). Similarly, the miR-93 level was significantly elevated compared to those without metastasis (Fig 1B). Moreover, the higher miR-93 expression was associated with advanced clinical stage (Fig 1C). To further validate the correlation, we monitored miR-93 levels in six gastric cancer cell lines as well. It was found that the expression level of miR-93 was significantly increased in MKN-28, BGC-823, MKN-45, MGC-803, SGC-7901 compared to the normal gastric epithelial GES-1 cells (Fig 1D). These results indicated that the expression of miR-93 was elevated in gastric cancer tissues and cells and may serves as a promoter in gastric cancer. Additionally, because the expression of miR-93 was highest in SGC-7901 cell line, while lowest in BGC-823 cell line, these two cell lines were used to in our study for investigating the molecular mechanisms.

### MiR-93 expression is associated with clinicopathological characters of gastric cancer patients

Because the expression of miR-93 was elevated in gastric cancer tissues, we next examined whether miR-93 expression level was associated with clinical characteristics. As shown in Table 2, the gastric cancer tissues and their paired non-tumor tissues from 70 cases were detected, and it was shown that higher expression of miR-93 was significantly associated with lymph node metastasis and advanced TNM stage (P<0.05). However, the expression level of miR-93 was not obviously associated with gender, age at surgery, tumor size, and histological type. These results suggested that up-regulation of miR-93 may be involved in progression and metastasis of gastric cancer.

**Table 2:** Correlation between clinicopathological characteristics and miR-93 expression.

Characteristics	No. of case	%	miR-93 expression(mean ± SD)	*P* value
**Age(years)**				
**<50**	17	24	4.934±2.253	0.632
**≥ 50**	53	76	4.682±1.751	
**Gender**				
**Male**	52	74	4.457±1.628	0.132
**Female**	18	26	5.196±2.163	
**Tumor size**				
**<5cm**	43	62	5.194±1.678	0.155
**≥5cm**	27	38	4.578±1.849	
**Tumor location**				
**Cardia**	16	23	5.258±2.371	0.496
**Body**	11	15	4.667±1.864	
**Antrum**	43	62	4.480±1.741	
**Differentiation**				
**Well**	6	9	3.413±1.456	0.138
**Moderately**	34	48	5.125±1.832	
**Poorly**	30	43	4.863±2.145	
**TNM stage**				
**Stage I-II**	20	28	3.439±2.068	**0.001**
**Stage III-IV**	50	72	5.274±2.135	
**Lymph node metastasis**				
**Metastasis**	45	64	5.250±2.256	**0.000**
**No metastasis**	25	36	2.854±1.392	

### MiR-93 regulates cell proliferation, apoptosis and cell cycle distribution of gastric cancer cells

**Fig 2:**
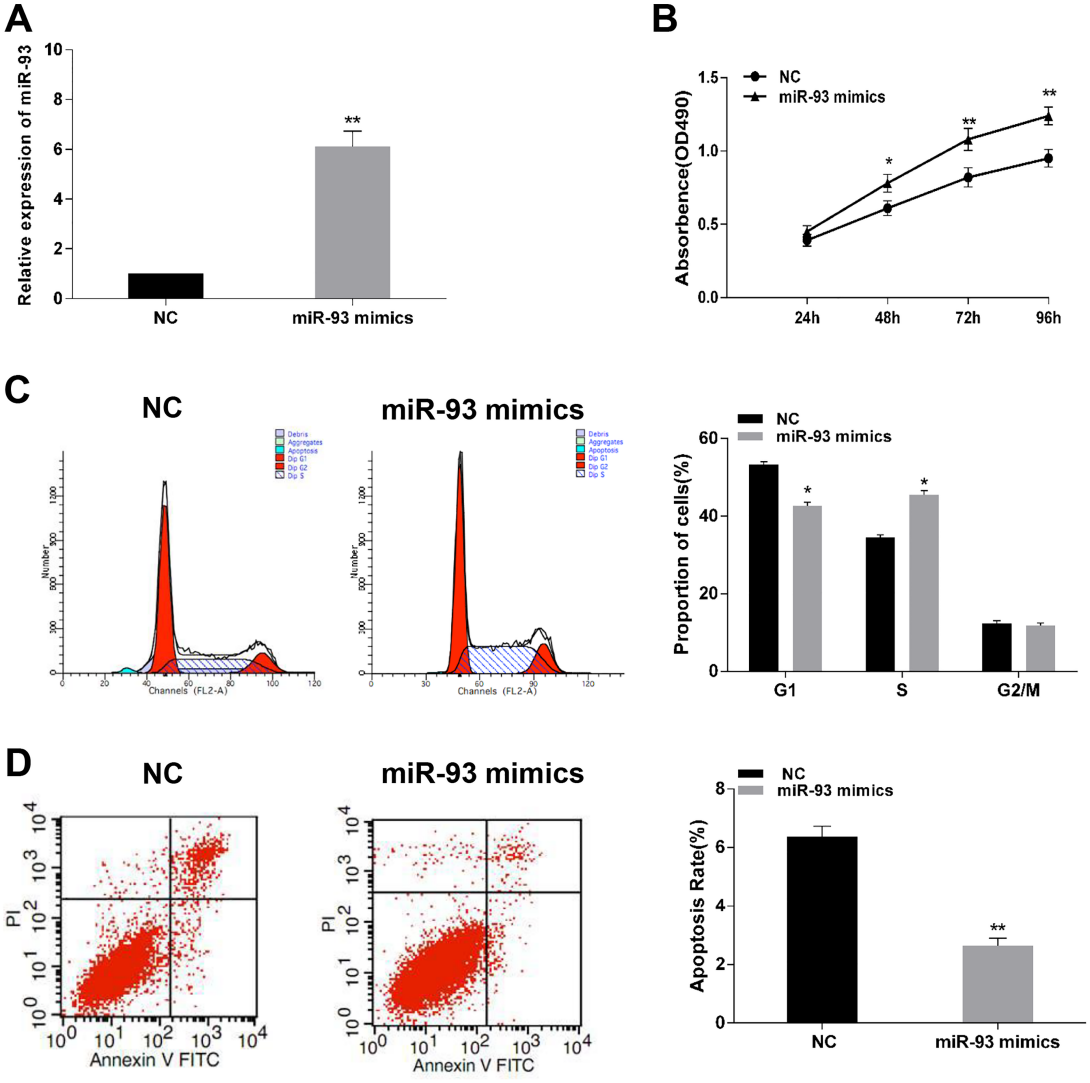
Effect of miR-93 upregulation on cell viability, cell cycle and apoptosis rate of BGC-823 cells. (A) miR-93 mimics significantly enhanced the expression of miR-93 in BGC-823 cells. (B) Graphical representation of MTT assay showed in BGC-823 cells transfected with miR-93 mimics or control. (C and D) Cell cycle analysis and apoptosis assay of BGC-823 cells transfected with miR-93 mimics or control. *P<0.05, **P<0.01.

**Fig 3:**
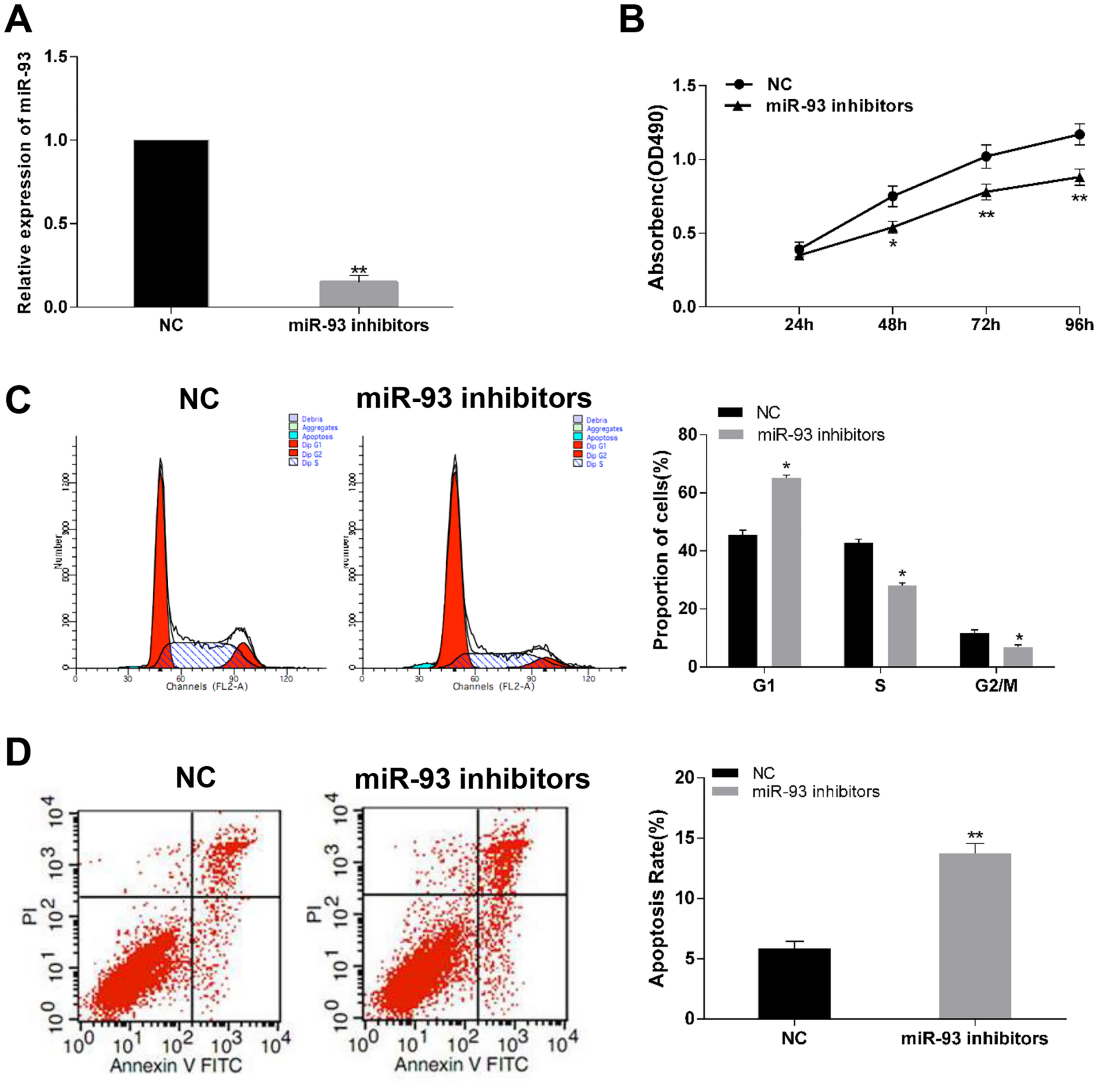
Effect of inhibition of miR-93 on cell proliferation, cell cycle distribution and apoptosis rate of SGC-7901 cells. (A) miR-93 inhibitors significantly decreased the expression of miR-93 in SGC-7901 cells. (B) Representative graphs of MTT assay showed in SGC-7901 cells transfected with miR-93 inhibitors or control. (C and D) Cell cycle analysis and apoptosis assay of SGC-7901 cells transfected with miR-93 inhibitors or control. *P<0.05, **P<0.01.

To explore the biological function of miR-93 in progression of gastric cancer. Gain and loss function assays were performed by stable transfection of miR-93 mimics and miR-93 inhibitor. RT-PCR assay showed that transfection of miR-93 mimics significantly increased miR-93 expression in BGC-823 cell (Fig 2A). MTT assay showed that overexpression of miR-93 dramatically promoted cell proliferation of BGC-823 cells (Fig 2B) compared to the negative control group. 48 h after transfection of miR-93 mimics in BGC-823 cells, the cell cycle distribution was investigated by using Flow cytometry (Fig 2C). Compared with control transfection group, the percentage of G1-phase was significantly decreased in BGC-823 cells. Following judgement of cell cycle, we performed PI and Annexin V double staining to investigate apoptosis induced by miR-93 mimics (Fig 2D). It was shown that transfection of miR-93 mimics decreased the percentage of apoptotic cell in BGC-823 cells compared with the control group. On the other hand, transfection of miR-93 inhibitor obviously suppressed expression of miR-93 in SGC-7901 cells (Fig 3A). Inhibition of miR-93 could suppress cell viability (Fig 3B), undergo cell cycle arrest in G1-phase (Fig 3C) and induce apoptosis (Fig 3D) of SGC-7901 cells compared with their negative control. These results demonstrated that up-regulation of miR-93 could accelerate cells proliferation.

### MiR-93 promotes migration and invasion in gastric cancer cells

**Fig 4:**
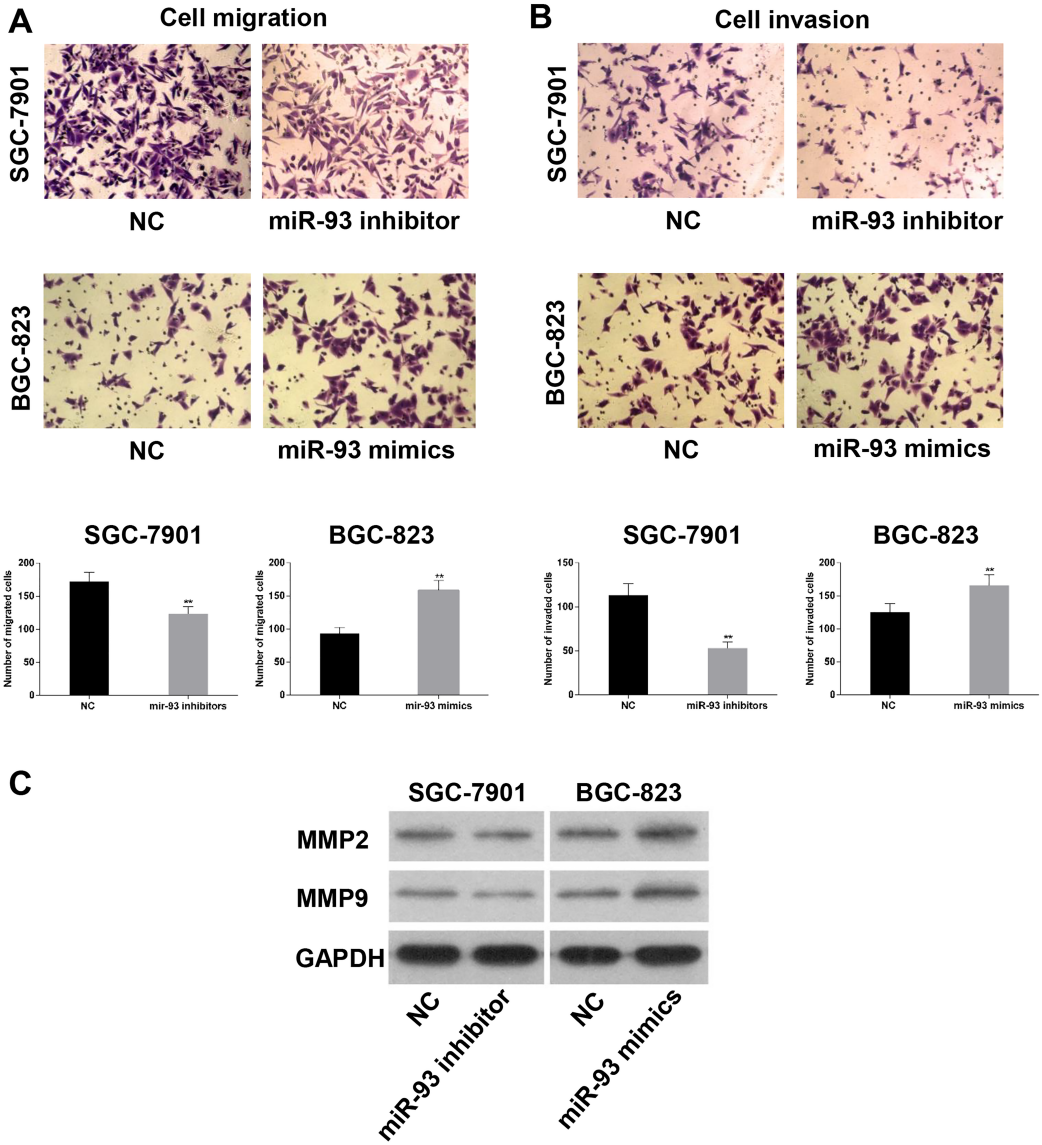
MiR-93 promoted cell migration, invasion and metastasis *in vitro*. (A and B) Ectopic expression of miR-93 in BGC-823 cells promoted cell migration and invasion, moreover, knockdown of miR-93 in SGC-7901 cells inhibited cell migration and invasion. (C) Levels of MMP-2, MMP-9 proteins in gastric cancer cells measured by western blot analysis, GAPDH was used as control. *P<0.05, **P<0.01.

Transwell assay was employed to assess the function of the miR-93 on the progress of gastric cancer. Transwell assay showed that compared with the control group the number of migrated and invaded BGC-823 cells was significantly increased after transfection of miR-93 mimics, while, knockdown of miR-93 significantly decreased the migration and invasion ability of SGC-7901 cells (Fig 4A and 4B). In addition, we also evaluated metastasis related proteins including MMP-2 and MMP-9. Western blot assays showed that expression of MMP-2 and MMP-9 were upregulated in mimics group, while the opposite results were obtained in the inhibitor group compared to negative control (Fig 4C). Therefore, we concluded that miR-93 served as a promoter on migration and invasion in gastric cancer cells.

### Effect of miR-93 on Xenograft tumor growth *in vivo*

**Fig 5:**
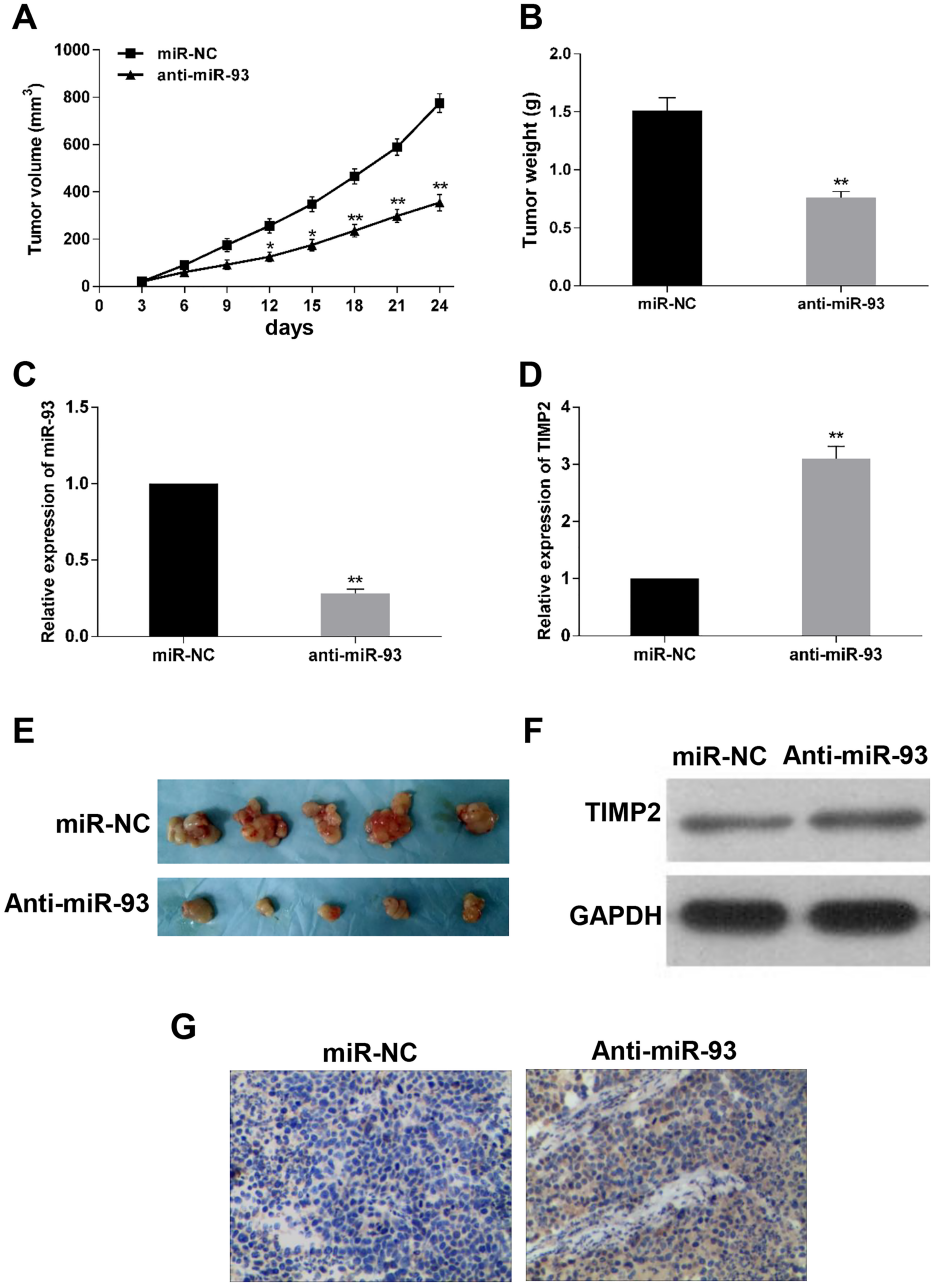
Antagomir-93 inhibits tumor growth *in vivo*. After transfection of SGC-7901 cells with antagomir-93 or antagomir-NC for 24h, cells were subcutaneously injected into the Left subaxillary of nude mice. (E) Representative images of excised xenograft tumors from nude mice. (A) The growth curve of tumors in nude mice. (B) Tumor weight. (C) Tumor levels of miR-93 measured by Quantitative RT-PCR. (D and F) TIMP2 miRNA expression and proteins levels measured in tumor tissues. (G) TIMP2 proteins levels in xenograft tumors determined by immunohistochemical staining. *P<0.05, **P<0.01, (magnification × 200).

To further verify the role of miR-93 in cells, xenograft tumor formation assay was performed to determine the effect of miR-93 on tumorigenicity ability *in vivo*. Hsa-miR-93 antagomir or the scramble control was transfected into SGC-7901 cells and then injected into the athymic nude mice. After tumorigenesis, we found that tumors in the miR-93 antagomir group grew much slowly and tumor size was much smaller than that of scramble control group (Fig 5A and 5E), and the tumor weight also reduced in the miR-93 antagomir group compared to the scramble group (Fig 5B). Moreover, we found that the expression of miR-93 was downregulated (Fig 5C), while both mRNA and protein levels of TIMP2 was upregulated in the anti-miR-93 group compared to scramble group (Fig 5D and 5F). In addition, we examined whether TIMP2 was negatively regulated by miR-93 using IHC staining, and found that the protein level of TIMP2 in anti-miR-93 group was higher than that in scramble group (Fig 5G). These results demonstrated that miR-93 may acted as tumor oncogene and promote tumorigenicity *in vivo*.

### MiR-93 is associated with epithelial-to-mesenchyme transition in gastric cancer

**Fig 6:**
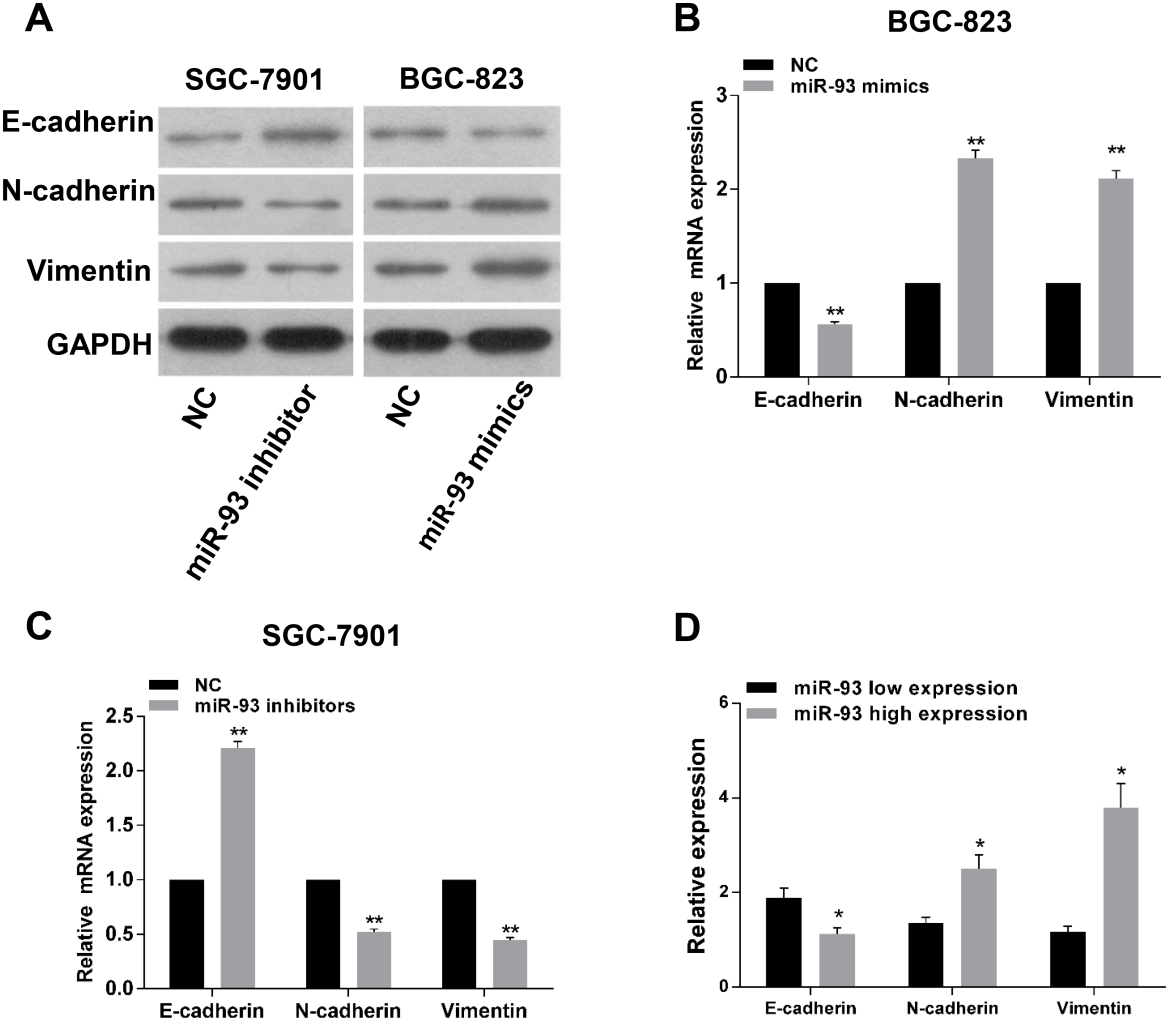
The correlation between miR-93 and epithelial-mesenchymal markers in gastric cancer. (A) The western blot assay showed that the EMT markers including N-cadherin and Vimentin expression were up-regulated after transfection of BGC-823 cells with miR-93 mimics. In contrast, N-cadherin and vimentin protein were suppressed after transfection of SGC-7901 cells with miR-93 inhibitors. (B and C) qRT-PCR analysis of E-cadherin, N-cadherin and Vimentin in BGC-823 and SGC-7901 cell lines. (D) qRT-PCR analysis of E-cadherin, N-cadherin and Vimentin in gastric cancer tissues.*P<0.05, **P<0.01.

To understand the effect of miR-93 in EMT, we transfected miR-93 mimics or inhibitor into cells to investigate the variations of EMT markers. Western blot assay showed that the expression of N-cadherin and Vimentin were significantly increased, and the expression of E-cadherin was decreased in the miR-93 mimics BGC-823 cells, whereas opposite results were shown in miR-93 inhibitor group (Fig 6A). Moreover, we also detected the expression of E-cadherin, N-cadherin and Vimentin in cells by qRT-PCR and got the similar results (Fig 6B and 6C). Because miR-93 could alter the expression of EMT markers in gastric cancer cells, we further detected whether this phenotypes also occur in gastric cancer tissues. To address this question, we detected the expression of E-cadherin, N-cadherin and Vimentin mRNA in gastric cancer tissues by qRT-PCR. The results showed that the expression levels of N-cadherin and Vimentin were significantly upregulated in miR-93 high expression group, while E-cadherin expression was downregulated (Fig 6D). These date suggested that miR-93 could induce EMT characteristics in gastric cancer. Notably, EMT is the most important initiation factor in the metastasis of tumor. Since transwell assays already indicated that migration and invasion were significantly increased after overexpression of miR-93, thus our results suggested that miR-93 promoted the EMT phenotypes, and then increased migration and invasion ability *in vitro*.

### TIMP2 is a direct target of miR-93 in gastric cancer cells

**Fig 7:**
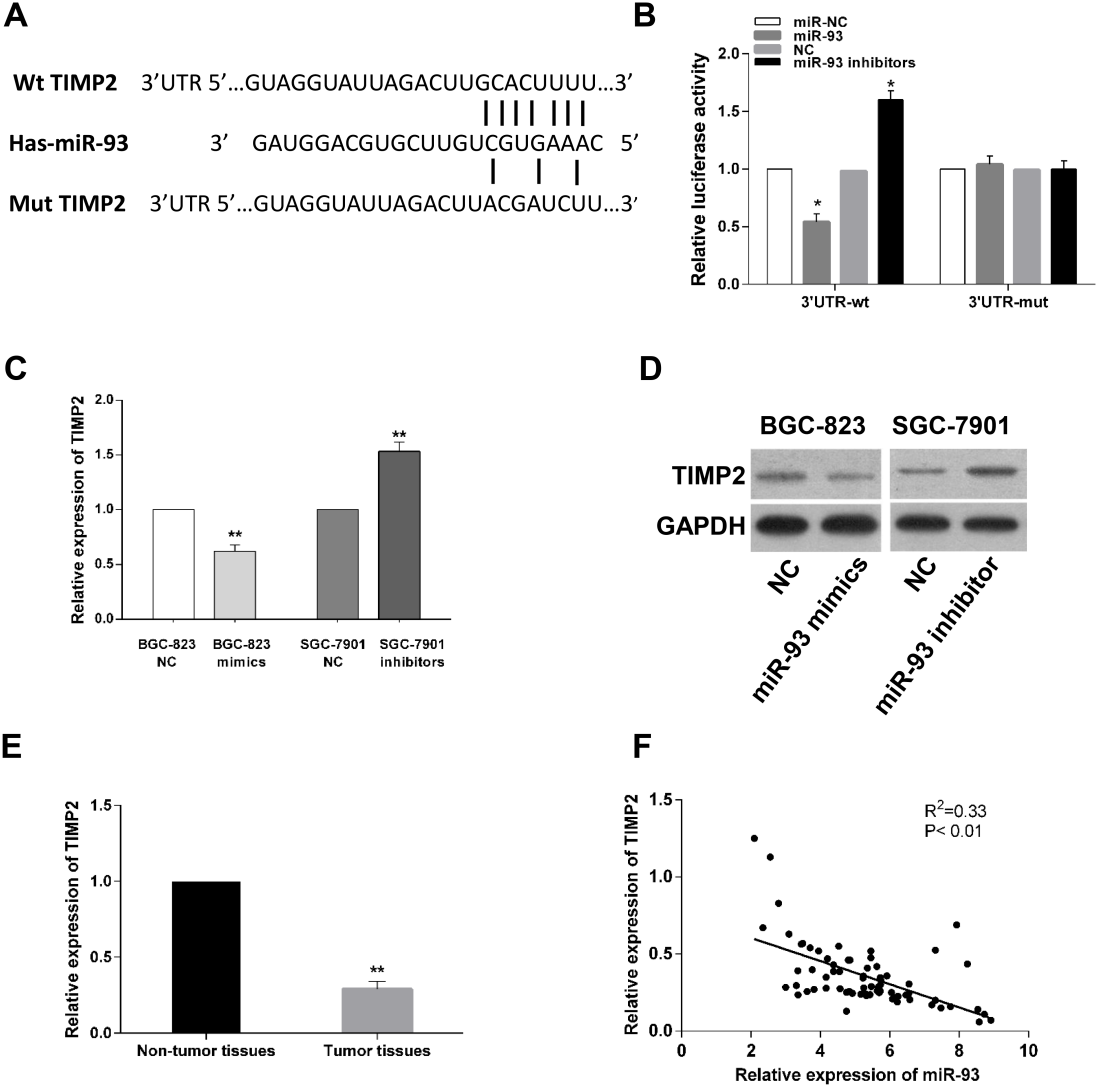
TIMP2 is a direct target of miR-93. (A) The predicted complementary sequence interaction between human miR-93 3'-UTR and TIMP2. (B) Up-regulation of miR-93 significantly inhibited while miR-93 inhibition promoted the luciferase activity of wild-type rather than mutant 3'-UTR of TIMP2. (C and D) Ectopic expression of miR-93 in BGC-823 significantly reduced the mRNA level and protein of TIMP2. Knockdown of miR-93 in SGC-7901 cells increased TIMP2 mRNA and protein level. (E) Relative expression level of TIMP2 in gastric cancer tissues and corresponding normal tissues. (F) The correlation between TIMP2 and miR-93 mRNA expression in 70 cases of gastric cancer specimens was evaluated using Spearman’s correlation analysis (P<0.01, R^2^ = 0.33). *P<0.05, **P<0.01.

Several bioinformatics softwares including TargetScan, miRDB, PicTar and miRanda were used to validate complementarity between miR-93 and 3’-UTR region of TIMP2 mRNA. Multiple active results indicated that TIMP2 has candidate binding sites with miR-93 (Fig 7A). TIMP2, which is a central player associated with cellular metastasis. We further performed luciferase report assay and found that overexpression of miR-93 significantly inhibited the luciferase activity of wild-type 3’-UTR of TIMP2, while knockdown of miR-93 significantly increased the luciferase activity of wild-type TIMP2 3’-UTR. Transfection of miR-93 mimics or inhibitor had no effect on the luciferase activity of mutant TIMP2 3’-UTR (Fig 7B). To further confirm the interrelation between miR-93 and target gene, qRT-PCR and western blot assay were performed to evaluate the effect of miR-93 on TIMP2 expression in gastric cancer cells. Results showed that both the mRNA and protein levels of TIMP2 in miR-93 overexpression group were significantly decreased compared with negative control (Fig 7C and 7D). In contrast, TIMP2 mRNA and protein levels were markedly increased in miR-93 inhibitor group. Furthermore, we also found the relative expression of TIMP2 was significantly decreased in 70 clinical gastric cancer tissues in which miR-93 was increased (Fig 7E), and the expression of miR-93 was negatively correlated with that of TIMP2 in gastric cancer tissues (Fig 7F, R^2^ = 0.33, P< 0.01). Altogether, we validated that TIMP2 was a direct target of miR-93.

### TIMP2 mediates the biological functions of miR-93 in gastric cancer cells

**Fig 8:**
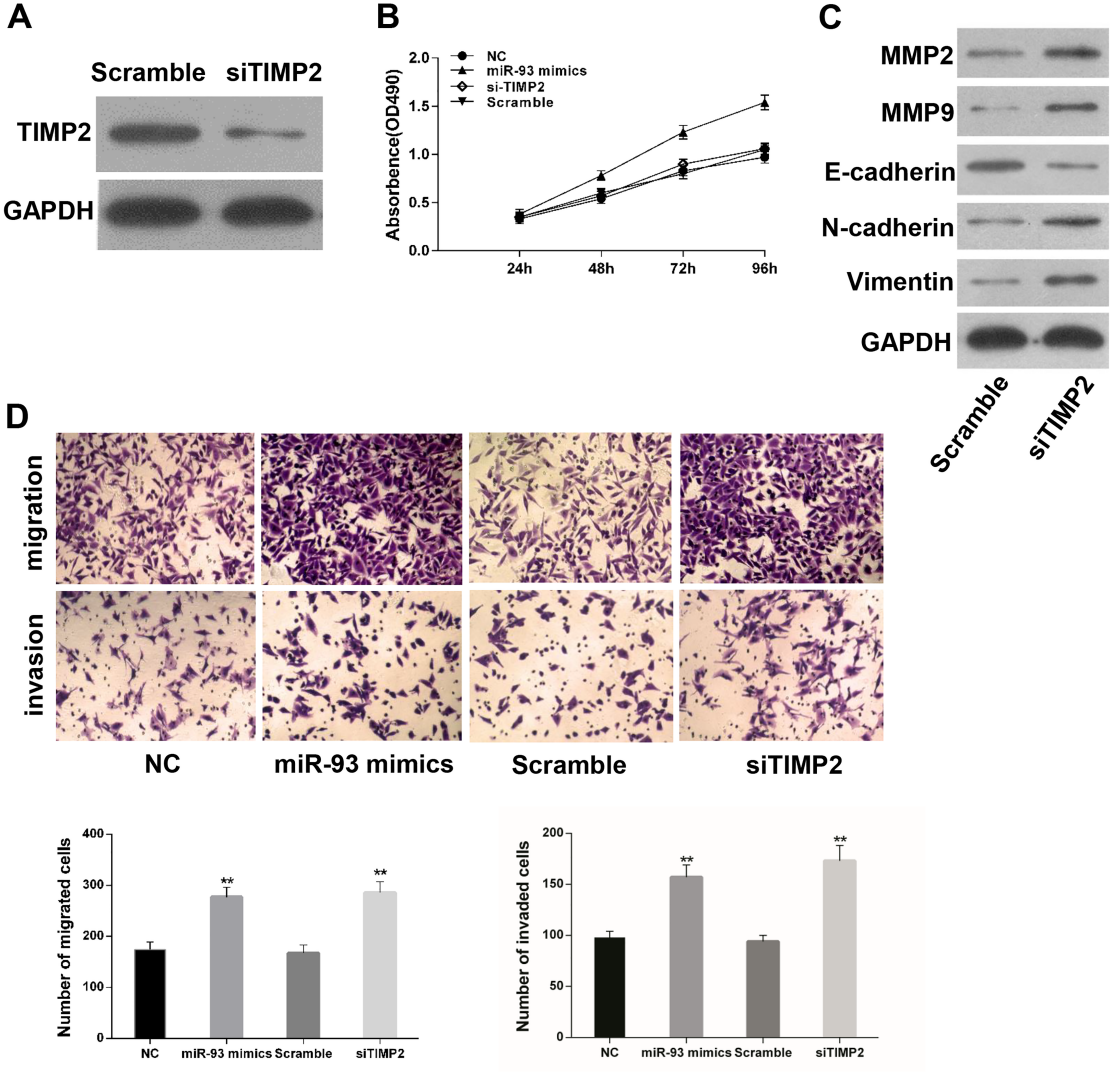
TIMP2 mediates the effects of miR-93 in gastric cancer cells. (A) TIMP2 protein expression was inhibited by siRNA. (B) MTT assays of SGC-7901 cells transfected with miR-93 mimics, NC, si-TIMP2 or Scramble. (C) The expression of MMP-2, MMP-9, E-cadherin, N-cadherin and Vimentin was evaluated by Western blot analysis. (D) Migration and invasion assays were performed in SGC-7901 cells transfected with miR-93 mimics, NC, siTIMP2 or Scramble. *P<0.05, **P<0.01.

To ascertain whether TIMP2 could regulate the biological role of miR-93 in gastric cancer cells. We transduced miR-93 mimics, negative control, siRNA-TIMP2 and si-Scramble into SGC-7901 cells. Transfection of si-TIMP2 significantly decreased the protein level of TIMP2 in gastric cancer cells (Fig 8A). However, si-TIMP2 has no effect on the proliferation of SGC-7901 cells (Fig 8B). It was speculated that miR-93 enhanced cells viability through other pathways instead of TIMP2. We further investigated the role of si-TIMP2 in tumor metastasis, and western blot was performed to detect the expression of MMPs and EMT related markers. It was found that MMP2 and MMP9 protein levels were dramatically up-regulated in the si-TIMP2 group. Furthermore, silencing of TIMP2 significantly increased EMT makers including N-cadherin and Vimentin, while decreased E-cadherin expression in gastric cancer cells (Fig 8C). Compared to the control group, transwell assays showed that silencing of TIMP2 significantly promoted cell migration and invasion ability (Fig 8D). These results suggested that TIMP2 could mediates the biological functions of miR-93 in progression and metastasis of gastric cancer cells.

## Discussion

Previous studies have shown that miRNA acts as promoter or suppressor, and is associated with tumor biological behavior in various human cancer [12–15]. Thus it is important to investigate the potential molecular mechanisms of tumorigenesis and pathogenesis mediated by miRNA in human cancer.

MiR-93 is located in the intro 13 of host gene Mcm7 at chromosome 7q22 [16]. Abnormal expression of miR-93 have been reported in several type of cancers, such as breast cancer [17], endometrial Carcinoma [18] and hepatocellular carcinoma [19]. Previous studies have demonstrated that miR-93 acts as an oncogene in cancers that can accelerate tumor growth, invasion and angiogenesis. In contrast, miR-93 was also reported to suppress tumorigenesis in ovarian cancer cells [20]. The opposite conclusion may be attributed to the fact that miR-93 play specific functions in differentiation degree and types of carcinoma. In addition, it was shown that miR-93 was also involved in the occurrence or progression of gastric cancer [21]. However, the biological functions of miR-93 and molecular mechanisms remain unclear in gastric cancer. In this study, we aimed to clarify the precise role of miR-93 in various biological processes and underlying mechanisms involved in gastric cancer.

In the present study, we observed high expression of miR-93 in both gastric cancer cells and tissues. Meanwhile, elevated expression of miR-93 was related to clinicpathological parameters of gastric cancer patients. In addition, we confirmed that ectopic expression of miR-93 potentially promoted cell proliferation, migration and invasion in gastric cancer cells, while repressed cells undergo apoptosis and cell cycle arrest at G1 phase. We further established the miR-93 knock down nude mice xenografts model and found that miR-93 could promote tumor formation in nude mice.

EMT has been demonstrated to be a crucial link associated with the progression of metastasis, and strengthens cellular migration and invasive ability. Many miRNAs have been reported to be involved in this process [22–23]. Through EMT process, the transformed cells lost their epithelial features and gained mesenchymal phenotype, which allowed these cells to access endothelial barriers and then entered the blood circulations and all parts of body [24–25]. In this study, we found that critical molecular markers of EMT were affected by miR-93, and the expression of mesenchyme cell marker N-cadherin and Vimentin were upregulated, while the epithelia marker E-cadherin was downregulated in gastric cancer cells. Therefore, we demonstrated that miR-93 enhances cell migration, invasion, and potentiate EMT in gastric cancer cells. Our *in vitro* and *in vivo* data together indicated that miR-93 serves as a promoter for tumor progression and acted as a predictor in gastric cancer patients.

To further characterize the potential action mechanism of miR-93 in gastric cancer, we employed bioinformatics software to search the target genes of miR-93 [26–27]. TIMP2 was identified as the top candidate target of miR-93, and was further confirmed by using luciferase activity assay. TIMP2 belongs to TIMP family genes encoding natural inhibitors of the matrix metalloproteinases, a group of peptidases involved in degradation of the extracellular matrix [28]. It has been reported that TIMP2 plays a role of suppressor in the regulation of cell proliferation and metastasis of malignant breast cancer [29]. TIMP2 was also reported to significantly inhibit invasion ability in glioma by negatively regulating miR-20a [30]. Another study of non-small cell lung cancer has proved that TIMP2 was a predicted downstream target of miR-761 [31]. In this study, we demonstrated that both mRNA and protein levels of TIMP2 were decreased after up-regulation of miR-93 in gastric cancer cells, but increased via anti-miR-93. Furthermore, silencing of TIMP2 enhanced migration and invasive ability of gastric cancer cells, which was consistent with the result obtained from the up-regulation of miR-93. Moreover, the expression of miR-93 was negatively correlated with that of TIMP2 in gastric cancer tissues. Taken together, our results provided evidence that TIMP2 is a direct target of miR-93 and mediates miR-93 promotion of gastric cancer progression.

## Conclusion

In conclusion, our study demonstrated that miR-93 was upregulated in gastric cancer and was associated with poorer clinical parameters in patients with gastric cancer. We further clarified that miR-93 functions as a promoter in cell proliferation and metastasis *in vitro* and tumor formation *in vivo* by direct target of TIMP2. Deciphering the molecular mechanism of miR-93’s role in human gastric cancer may advance our understanding of pathological mechanism underlying human gastric carcinogenesis, and provide a theoretical basis for further research in early diagnosis, clinical classification and biological therapy.

## Supporting information

S1 Fig The uncropped blots for the western blot results. (TIF)

S1 Table Tumor volume of mice in scramble and anti-miR-93 groups. (XLSX)

S2 Table Tumor weight of mice in scramble and anti-miR-93 groups. (XLSX)

S3 Table The underlying data points for Luciferase report assay. (XLSX)
