# Supplementary figures and images for: MicroRNA-93 promotes proliferation and metastasis of gastric cancer via targeting TIMP2
Source: PLoS One. 2017 Dec 8;12(12):e0189490. doi: 10.1371/journal.pone.0189490 (PMC5722343; doi:10.1371/journal.pone.0189490)

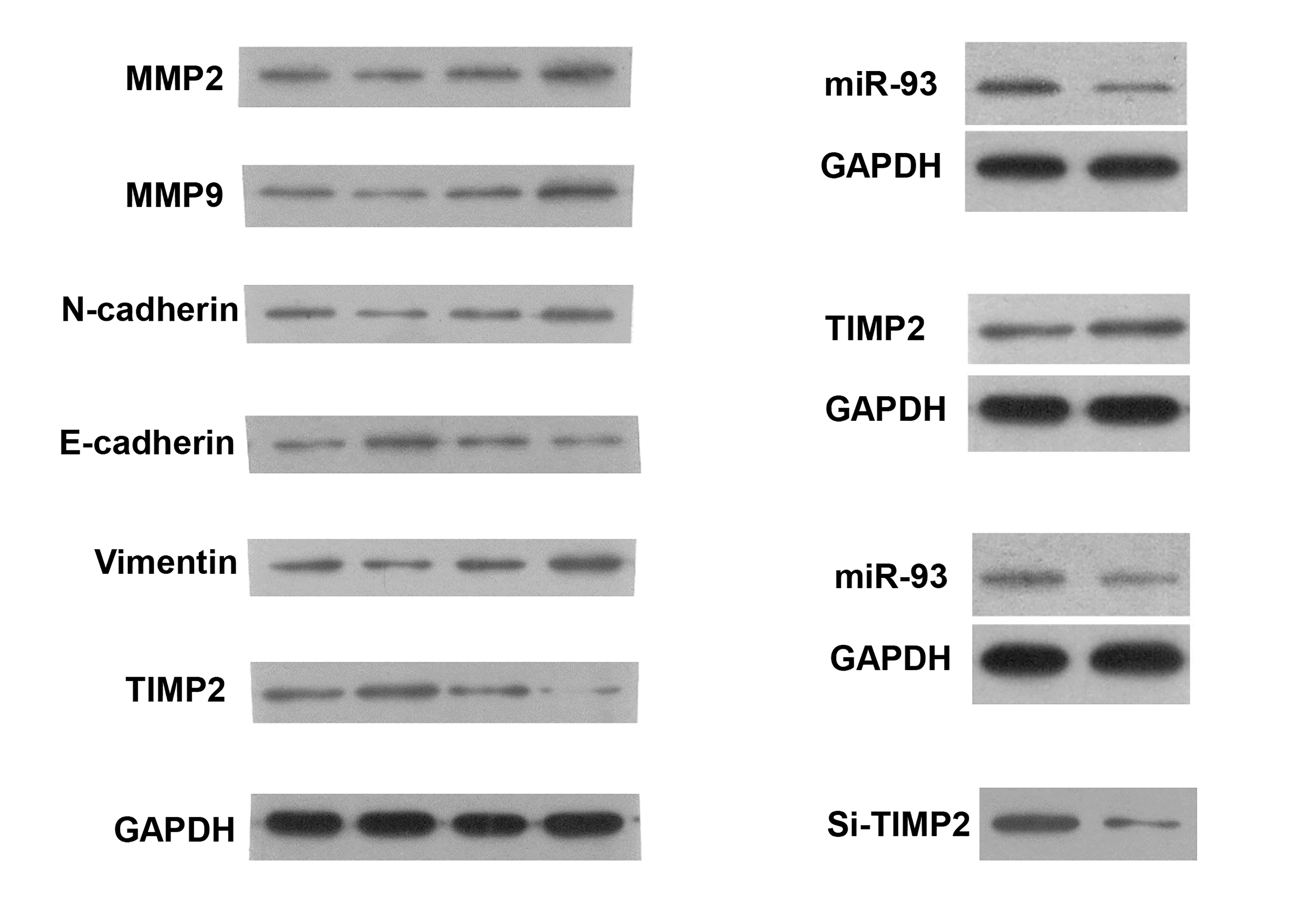

Supplement: S1 Fig — (TIF) [file pone.0189490.s001.tif]
